# Navigating neurophotonics, words of wisdom: an interview with Professor David Kleinfeld

**DOI:** 10.1117/1.NPh.9.1.010401

**Published:** 2022-03-28

**Authors:** Nozomi Nishimura

**Affiliations:** Cornell University, Meinig School of Biomedical Engineering, Ithaca, New York, United States

## Abstract

David Kleinfeld, the Dr. George Feher Endowed Chair in Experimental Biophysics at UC San Diego, shares his views on training and best practices in neurophotonics, offering words of wisdom for building and strengthening our scientific community.

**Figure:**
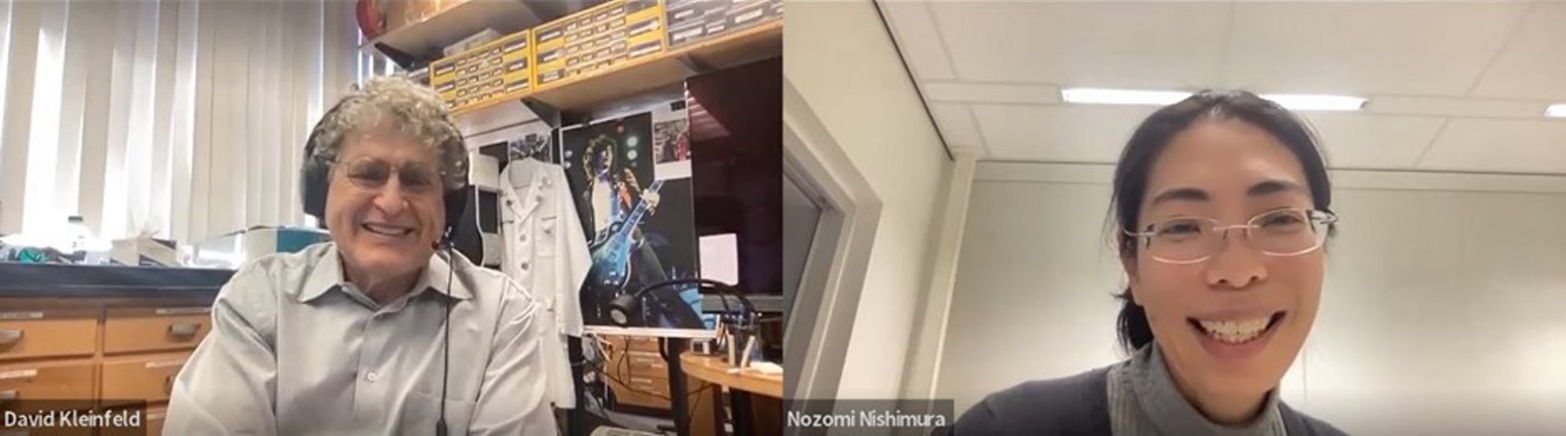
Neurophotonics *associate editor Nozomi Nishimura interviewed David Kleinfeld, the Dr. George Feher Endowed Chair in Experimental Biophysics at UC San Diego. Readers are also invited to enjoy the interview video at*, https://doi.org/10.1117/1.NPh.9.1.010401.

Nozomi Nishimura: Hi everyone! I’m Nozomi Nishimura I am currently an associate professor at Cornell and I’m going to talk to my former graduate advisor David Kleinfeld at UCSD about what it’s like to be at the interface of optics and neuroscience, like in neurophotonics. David, thanks a lot for doing this!

David Kleinfeld: Thank you!

Nozomi Nishimura: So, I really wanted to ask you about some tips, especially for people coming through the training now who want to be in both optics and neuroscience, or somewhere at the boundary of these kinds of things. From my own experience coming up in your laboratory—your laboratory does these things, both the optics and the neuroscience—so I want to know, what’s the “secret sauce” for doing that?

Maybe we could start with what you did in the past, because I don’t think you started in neuroscience right? What did you do before?

David Kleinfeld: I started my research career in a laboratory run by a professor named George Feher, here at UCSD. But I’ll backup considerably—I started in electrical engineering at University of Illinois (UIUC), which I enjoyed until I realized halfway through one course that all the homework was “exactly” the same. I mean somehow we were using the convolution theorem for one week, then two weeks, then three weeks—too much! So, I decided it was time to move on. My next year was spent as a work-study student. I was at Argonne National Laboratories, and there was a fellow who ran the proton injection beam by the name of James Simpson. He probably has no idea I existed. But Jim knew how to do everything, and he was a physicist. I decided that when I went back to school, I too was going to be a physicist.

Back at UIUC, I worked as a machinist for a while the laboratory of a professor named Hans Frauenfelder, who was a very strong physicist and was looking at molecular motions in the protein hemoglobin, and it was kind of cool! They used optics—at the time I wasn’t thinking of optics—but it struck me that there was just a world out there where you could look at biological problems using physics thinking and physics tools. Hans was pushing me to go to San Diego for a PhD and contacted George on my behalf, which was very kind of him. There was also another professor—Lorella Jones, who taught my field theory class—and she too was urging me to go out to La Jolla. And not only for science, at least because I was climbing in those days and she was saying this would be a great place to go.

So, I went to UCSD and joined George’s laboratory. His was basically a condensed matter laboratory, but we looked at a problem from biology, which was the conversion of light into chemical energy across the membrane in this photosynthetic protein. It was very fundamental. I had to learn a little bit of biochemistry, which is not the most painful thing in the world—Nozomi, you probably also do a little bit of biochemistry. The people I hung out with in graduate school were, for the most part, condensed matter experimentalists.

The thing that got me into neuroscience, and I think what got a number of people into neuroscience, was a paper on attractors in neuronal networks by John Hopfield that came out in 1982. Now I knew zero about neurobiology at that time. But John wrote this paper that made connections between spin systems, which was something a lot of my peers as well as senior colleagues were working on, and it seemed fascinating. And I was in a sports club at the time and had a mathematician friend who also got taken in by John’s paper, so we gave some informal graduate lectures on it. And then I started sniffing around for postdocs, having no clue what this entailed.

I think this is often common: you hear a talk, you read one paper, and somehow you learn something new that gets you going. You’re not sure why, and you don’t know anything about the field. But there is some attraction to an idea. I would say, even to this day when I am teaching things related to this early neuronal networks paper, it’s not clear what that paper has to do with any nervous system *per se*. But it is clear looking back that John’s paper ignited a little revolution and attracted many more people to neuroscience than just me.

Nozomi Nishimura: Fair enough.

David Kleinfeld: I didn’t know anything about finding a postdoc. The first mistake I made was not talking to anybody local, not visiting a few neuroscience laboratories at UCSD and asking—anyone—“What should a young physicist do if they want to go into neuroscience?” I might have gotten some good advice, or not. I just wrote to a number of laboratories based on who was well known and who was recommended by more senior friends who were already professors at universities. Only one “big name” wrote back and in retrospect I’m very happy that none of these worked out.

The first, albeit obvious lesson is that writing cold to people often doesn’t work—you need to have somebody sort of pave the path for you. I think that’s common. It’s not like you get ahead because of who you know, but if a trusted colleague comes up to you and says, “I know this young kid and they did blah blah blah, and it seems to be very good work,” usually—since you want to get the best possible graduate students in your laboratory or postdocs in your laboratory—this really helps.

That leads to the second lesson: it’s good to know a lot of senior scientists, even when you’re in graduate school or as a postdoc, because these are people who could help you make decisions and can help you pave the way forward. I should have used this resource more effectively. In the end, at least two people got me interviews at places with strong laboratories. That was great!

The critical recommendation came from the late Phil Platzman, who was a senior scientists at Bell Laboratories and regularly visited UCSD to work with the late Prof. Sheldon Schultz. Shelly was a mentor to many of my friends and would go on to discover metamaterials. Phil was always recruiting during his visit and this was the era where the place to go if you’re in condensed matter physics was Bell Laboratories. It was the most famous place on the block. Phil came around and was kind of talking up Bell and three of us went out there: Donald Eigler, who became famous for writing “IBM” in atoms and who started the nanoscience revolution; Eric Gullikson, who was a close friend, a close sports friend too—Eric was a great cyclist and now works at Lawrence Berkeley Laboratories, and myself. I heard that they were starting a neuroscience group at Bell and that John Hopfield was associated with this. I mean, how much better could it be, right?

So, for young people, this means just to meet and keep in touch with speakers and visitors coming through from other universities. Lucky breaks occur after you get to know people. I think that’s important. There’s always colloquium visitors and seminar speakers coming through, even if recruiters are maybe a little less popular these days.

Nozomi Nishimura: Yes, talking to people—that’s what I remember about your laboratory, too, that within the laboratory there were a lot of people that had all sorts of expertise and very friendly for just asking basic questions, and I think that was helpful. But what did you do—did you have anything like formal classes, to ramp up on the neuro side?

David Kleinfeld: What I think I should have done, even to this day, was to at least have sat in on a class. I should have found an interesting professor and just sat in the back of the class and listened in and tried not to ask too many questions. I didn’t do that, but I did apply to the summer neurobiology course at Woods Hole. There’s a couple places in the country where there are summer schools. I was fortunate. The course that accepted me was a 9-week program at the Marine Biology Laboratory. It was really a very classic course. It was the last year that this course—it was really sort of the Harvard neuroscience program—was taught in a classic manner. It was a little bit premolecular in style, but it was everything, from learning about single cells to the development and organization of nervous systems. There were morning lectures, afternoons in the laboratory, nights in the laboratory. At the very end of the program we had an anatomy section, which was really great. The anatomy part was taught by an NIH laboratory chief named Thomas Reese and we had a lot of fun. I learned to decorate cells with tiny (10 nm) metal balls. I even drove down to New Jersey for a few days in the middle of the class to use what was the then relatively new technique of scanning tunneling microscopy to look at these samples.

This brings up lesson number three. There’s a fair number of graduate and postgraduate summer programs and it’s something that I think all students should think about applying for.

As you know, as in your own case and Chris Schaffer’s case, there’s a fair number of former physicists who are doing neurobiology. If you look across things like the NIH BRAIN program, a flagship program from the NIH, they’re disproportionately populated by former physicists. This is not an accident, right? If you take biology seriously, being trained as a physicist is a good background.

Nozomi Nishimura: What about going the other way? You’ve had a lot of trainees that have come through neuroscience programs and things as well, and then you’ve taught—was it Woods Hole or Cold Spring Harbor—optics classes. What’s the best way to go the other way?

David Kleinfeld: The best way is maybe something that’s happening now. Cold Spring Harbor is famous for its summer programs in addition to Woods Hole. They have multiple 6-week programs. There’s one in imaging, which really turned into a nonlinear optics class. As a student, it’s total immersion—I believe in these things—and it’s a good class. I think a lot of people came out of that class and became familiar enough with microscopy that they were able to set up 2-photon and other systems in their own laboratory.

Most people trained solely in biology look at an optical bench and get nervous. They get nervous if the PI goes on vacation because if they turn a mirror the wrong way they’re worried that they’re not going to turn be able to turn it back. But I think the classes were very good at teaching kids fundamentals. I don’t know how many people out of all those classes we taught decided to say, “Oh I like this better than neuroscience and I’m really going to become some sort of an optics jock.” I think the courses were most effective at making people thoughtful about how they do their measurements and on removing all fear—or at least a lot of fear.

The best way forward for training, if it’s possible, might be something that’s happening now with a young graduate student in my laboratory. He came from neurosciences and his thesis project has to do with two oscillator networks that are in the brainstem. This is a tough place to record from—OK, it’s actually not as tough as people think although this perception has scared off a lot of our competitors—which we’re very happy about—but we want to take a shot at optically recording from cells in the brainstem. So one postdoc in the laboratory—a very talented postdoc, Rui Liu—built an adaptive optics 2-photon microscope, using labels in the bloodstream as a guide star. And we wanted to build a second system and this bold graduate student, Pantong Yao, just said, “I’m gonna do it!” He really didn’t know any optics when he came, but Rui was here to help, and I’m here. We already had a lot of material that was put together and we had Zemax programs to help with planning. And Pantong just put the thing together from scratch. He made a lot of mistakes—and then of course we found out that maybe we made some mistakes and had gotten lucky in places, but in the end it works and now he is an expert.

So as the fourth lesson—maybe the best way to learn if you are a trainee that has come through a neuroscience programs—is just to go to a laboratory, such as yours and Chris’s laboratory, and put something together. Maybe you have to spend a little extra time in graduate school, but at the end you have a kind of freedom, right? Because you know how to build things. I don’t think that’s crazy. Probably this was done in other laboratories and I’m just not aware of it, where people went in and said, “Okay, there’s a device out there, it’s not trivial, I’m going to assemble it myself and put myself through school.” And hopefully their advisor has some patience, because as you know it always looks simpler when somebody has these beautiful CAD-CAM diagrams in their paper.

Nozomi Nishimura: Right, well it’s interesting that you’re saying that a lot of it is just getting over the hesitation and diving in. I’ve always thought the main thing that—well, maybe not the only thing—but one thing that you do learn as a physicist is to—I don’t know—maybe be arrogant about it and just to go for it. And that maybe is the most useful thing!

David Kleinfeld: I think it is important. Such is physics. You train and train and you just do it, much like if you do sports—sports is a good analogy to doing science. But you also have to be realistic about your capabilities, and you have to put in the time. And like you say, “you just go for it.” I think the only difference—at least in physical sciences training—something that’s important is that you think about the feasibility of your experiment in fundamental terms. You learn to sit down with a pencil before you start and ask yourself, “Is this feasible, from a signal-to-noise perspective, from a materials perspective?” That’s something Phil Tsai and I tried—I don’t know how successful we were—to do at the Cold Spring Harbor Laboratory imaging course. Someone says, “I’d like to measure X,” but then you know something about the tissue, about the scattering, you know how many photons you can put in, you try to do a back-of-the-envelope calculation, which is probably the best that you ever could do to estimate if it’s feasible. But beyond this additional calculation, I think it’s just putting in the time and just sort of losing fear.

Nozomi Nishimura: That’s a good message. If I could kind of switch directions here: when I worked with you, 2-photon microscopy was sort of new and up-and-coming, but now there’s a lot of commercial 2-photon microscopes and 2- and 3- photon microscopy is showing up in a lot of neuroscience labs and is becoming more of an established technology. Any hints on what’s next on the horizon? Or, what should people at this neurophotonics interface be investing their time and training in?

David Kleinfeld: Okay, well, so one thing I have a little bit of a bias in—alright, we all do—is that you want to try to image as deep as possible. Or maybe more importantly, you want to try to stay at theoretical resolution, because the structures you’re looking at are really on the order of the wavelength of light. Right now, people are measuring neurons—that’s popular. As you say, at this point everybody and their brother or sister is measuring from neurons in layer 2-3, that’s plumb in the middle of processing in neocortex. You’d like to go down and look at the input, layer 4, and you’d like to look at the output, layers 5 and 6. Critically, from a theoretical perspective, you really want to be able to look at the synaptic connections. You really want to get an idea of how many inputs are actually active. How many different ways can the input arrive and activate the same output, which means you need to start looking at synapses. The parts of imaging that we’re dependent on are people making good tools, molecular tools, to look at synapses—but that’s sort of maturing now, I mean indicators of glutamate are getting very mature. I think of Kaspar Podgorski (Allen Brain) who’s been pushing this, among other people, for iGluSnFRs—GABA-sniffers are also coming along. Anyway to get to this resolution, you really need to use adaptive optics. That’s a little bit of a bias, as I mentioned it’s something we’re doing, but that’s something that many laboratories in the world are doing. It’s still pretty young, I mean there’s only a few commercial deformable mirrors out there that are available. Maybe two companies—there’s Alpao in France and there’s Boston Micromachines. They’re good, but the mirrors are insanely expensive at the moment, and delicate. But this I think has to become standard. Better ideas for guide stars need to be out there. So that’s a growth industry. That’s one, I mean it’s sort of an obvious one, but that’s one.

The other thing is something that we don’t do, but I think is important, and—while I don’t know everybody in the field anymore, Alipasha Vaziri at Rockefeller is pushing this—is to sort of mix in computational methods with scanning. In an ideal world, you’d like to randomly sample in some fashion—but maybe not completely random as is suggested from compressed sensing since you know something about the tissue. That’s not quite possible in nonlinear imaging, because the beams have significant spectral bandwidth and it’s hard to push the points around without getting a lot of aberration, but still you can do some sort of partial sampling based on priors, your knowledge of the anatomy, and maybe your knowledge or your biases towards which cells in your field are active. There’s a few examples out there where it’s done in a principled way. I think there’s a lot of opportunity in this direction. Also, you don’t really see the image until the end of the day. It’s not so different from in confocal, where until you do a maximum reconstruction, you don’t really see the beauty of your structure, so the imaging field already has a flavor of computation built into it.

It would be interesting to know what other people think, but that’s what I see as two growth areas now. So maybe this is a golden opportunity for people who have a mixed interest in technology and biology at the same time.

Nozomi Nishimura: Sounds really good, yes. You’ve always had biophysical modeling as well and thinking about the circuits from a computational point of view as well—so where does that fit in, the truly computational side?

David Kleinfeld: We do this—okay this is a little bit off optics. The other part of the laboratory which has been going on—it started with circuitry and then had to do with decoding whisking signals. I think sometime after you and Chris left, we had another great graduate student, Jeffrey Moore, and we had a wonderful visitor, Martin Deschênes, and we were able to make a lot of progress on the circuitry that drives whisking. We also picked up another wonderful collaborator, Fan Wang. Anyway, to fast-forward, this effort, which turned into a group effort, got to the point where we think we understand the circuit. So, rather than little dabbles toward modelling, we finally reached the point where we had to come clean. Working with a theorist in Israel, David Golumb—somebody I’ve known for 30 years—we put the bulk of the data together in a consistent picture. It was actually a very valuable effort. It fed back on a number of experiments now done in Fan’s laboratory and it gave us a little bit of a perspective.

I think it’s an advantage to come from physics, or to come from mathematics: you can do proper mathematical modeling—not just do some kind of naïve computer model. If you actually did go to class and learned your stat-mech well and learned mean-field functions well, you could do things where at least you have some very brutalized abstract form of what you measured, but you know what the limits are, you know the phase space of your solutions, etc. So that goes on in our laboratory. I have to say that the only place it really connected with the imaging was stuff that you worked on. You and Chris were here when we really started getting into blood flow seriously. We finally were able to put together—where we is graduate student Xiang Ji, having measured every capillary in the brain and the connections between them—a model connecting flow and energetics. Our analysis was accomplished using just classical knowledge—that is, first-year graduate student course work in physics—to put together the right diffusion equation.

Again, I really feel strongly that the best way to become a biologist is to study physics. Because you know in a sense what data you need to take in order—in principle—to make some simplified model. Then if things work out—if you’re lucky enough that things work out—then at least you can pull that model off by yourself, and if you can’t pull it off by yourself, then at least you know enough to talk to some experts to work with you. I think you need to convince all the high-school seniors who want to become biologists to major in physics when they go to college.

Nozomi Nishimura: I feel like there were some things that were useful, but I can think there are other ways, especially for someone fundamentally interested in the brain to come up and learn some of the measurement science and the quantitative things.

Those were really interesting stories to hear. Making another switch now, more on the technology side. For a while, the optical imaging and the calcium imaging were sort of taking off and it seemed like electrophysiology was sort of being left behind, but now with the Neuropixels and all of these arrays of recorded things, it seems like it’s sort of head-to-head. How do you think about, if you’re looking at some of the neuroscience questions, when do apply which tools? When do you go for an optics tool, and when do you go for electrophysiology which you’ve done a lot of as well? How do you pick and choose? It’s hard to become an expert in all of these things.

David Kleinfeld: That’s a fair question. They have different strengths and different problems. Neuropixels were conceived by Timothy Harris—who actually was at Bell, yet I barely knew him back then. Tim and I have become friends and Tim gets tremendous credit for pushing Neurpixels to production. But there’s an unsung hero at IMEC—the foundry in Leuven where they manufacture the probes—who when asked to improve the surface area, that is, the capacitance of the electrodes, started using titanium nitride. This is the stuff you put on drill bits in order to make them tough. Titanium nitride makes these little, tiny crystals that increase the surface area, and they don’t fall off, like occurs with platinum black. This is what makes each of the individual electrode pads have a very high surface area and a concomitant relatively low noise. That’s the underlying technical secret for how this stuff works so well. So, whoever she or he is, they should be given a small trophy or something.

Neuropixels has a smart design. It has good fundamentals, like I mentioned, in that it was packaged in a convenient way, with built-in electronics. What something like Neuropixels allows you to do is measure from many areas in the brain at once, and measure from parts of the brain that are just inaccessible with optics. At some point, with 2-photon or 3-photon absorption, there’s a scale—maybe about 2 millimeters—at which the light becomes a cloud and you’re not going to be able to make measurements. So, much as there are people pushing tricks to use acoustic modulation or other approaches that will take you maybe a little bit deeper, they’re generally not near optical resolution. So I think, short of some very new idea, you’re still imaging into a kind of a shell on the outer part of the brain, or you can take a vacuum cleaner to part of the shell and go deeper. I mean, that sounds terrible, but it works. All measurements have side consequences.

So, the Neuropixels work. It, like all techniques, makes use of an inference. You’re outside of the cell. You’re inferring what’s going on from statistical measure, saying there was a spike and I believe the spike only came from one cell. You’re forced to make certain assumptions in your analysis as to what the contamination level is. I think this part of the game has to be much better taught within the community. There’s a kind of sloppiness in the community about measures that require statistical inference. Neuropixels, again because of its capacitance issues, allows you to see the same spike on many electrodes so you always get a set of signals that appears like a point spread function, just like you get in optics. So that’s a benefit for the analysis. There’s a lot of fundamentals that happen with Neuropixels that are an improvement. But the big thing is you can record across areas in the brain—and maybe it’s gotten people to learn their anatomy a lot better because they have to pick a trajectory that puts the Neuropixels probe through a number of interesting regions. But you are making a trepan hole in the head, and you are putting a big spear through a lot of tissue. There’s been new science that’s come of it, right? I mean it’s sort of revived the idea that it’s not just anatomical connections that span almost the whole brain, but signals span almost the whole brain, even if they’re not directly contributing to the behavior at hand. It’s made experimentalists come to grips with the realization that you can’t look at a small area, that the key difficulty—or you might say, the key challenge—in the brain is that the connectivity and the signal spread is so extensive. So that’s one direction where an electrical tool is cannot currently be replaced by an optical tool.

Since we’re on the topic of electrodes: the other thing that a lot of us lament is that there is no better information than having an intracellular electrode in a cell. You’re listening to all the synapses. You can control the cell—you can turn it on, you can turn it off, you can change the reversal potential, due to mainly chloride, to see who’s an inhibitor at input. This skill is somewhat getting lost, and I think a lot of people would spend any amount of time measuring a calcium signal or something else besides learning classic electrophysiology. This isn’t trying to sound like some old-timer—you know, “Back in my days, we used to …,” but it’s really sort of a serious issue because there’s information that you can’t get if you’re not intracellular. That’s something universities really should take it upon themselves to teach all incoming graduate students. It’s just so valuable. Even if you only want to calibrate your electrodes, like Neuropixels, or calibrate your calcium signals—which are notoriously unreliable—you should be having an electrode in a cell. And I can count two people in the world who have done these dual measurements of electrodes and optical signaling at the same time. So that’s a little pitch.

I’ve had the pleasure of Martin Deschênes—who I mentioned earlier—who’s one of the gods of electrophysiology. Martin could record from motoneurons in an awake animal and hold the cell for 20 seconds—that was 200 whisks, which is a lot of data. Very quickly we found out we had an inventory of inputs from this one motonucleus, and we could delimit a circuit. I mean, it’s awesome! Awesome! No opto-this or opto-that, just straight measurements.

Back to optics. Neuropixels allows you to record in many places, but it doesn’t allow you to move around. You have to pull the probe out, put it back in. You are causing damage—you are slicing up axons. At some point, you’re going to start influencing behavior. You become a little bit suspicious about what’s going on. Optics allows you to move around. There are various ways to make very thin skull windows so you’re not really damaging the brain—, so you’re not changing the volume of the brain or the pressure in the brain. Patrick Drew and Phil Tsai—who I also mentioned earlier—came up with clever one while they were in the laboratory. But optics still has its limitations now. I mean, people are looking primarily at upper layers and signal flow, crudely, in cortex is inputs coming to anatomical layer 4 as well as anatomical layer 5, going up to layers 2-3, and going back down to layer 5. So, we know an awful lot about what’s happening in these middle layers, but we don’t really have a very holistic view of signal flow in cortex, much less with other pathways—colliculus pathways, cerebellar pathways. Even though there’s a huge number of papers coming out, in some sense it’s still an immature view of cortex because it’s not taking a holistic account of signal flow in the brain. I think that’s part of the opportunity. Whatever it takes—we’re a little biased towards adaptive optics now, but whatever your trick is, whatever your capability is, push the methods to really think first about signal flow. Then do whatever you can to measure signal flow throughout an entire structure.

Nozomi Nishimura: Yes. With optics, I was going to say that in combination with some of the genetic tools and things like that, that’s one of the advantages that we have. You can use promotor driven expression on a particular cell type or use some of the tracing tricks and things like that, so there are ways that you can get at some of the anatomy and more specificity. But—for the most part—you are limited to where you can see.

David Kleinfeld: Absolutely. If you did sparse labeling in a deep layer, even though you’re looking at a fuzz ball—very nonoptimal from the point of view of imaging science—you’re still answering your question, your scientific question. Yes, no doubt about this.

That actually brings up one other issue—to extend the question a little bit as there were two things you brought up. You have to know something about promoters. And you have to know something about how one can make a transgenic animal, and you have to know where you can actually cause additional expression without changing the physiology of the animal. Many of the mice that had expressed genetic calcium indicators in neurons ended up becoming epileptic because the indicator was strongly buffering calcium. You just can’t purchase a mouse out of an online catalog and think, “Oh yeah, somebody said it does so-and-so”—you could waste a lot of time in your life.

Another thing you bring up, which is very important, is indicators—there’s another world of people who are pushing indicators, who are looking throughout nature for more interesting proteins that they think they could contrive to modify cell function or report cell function optically.

These three communities—genetics, indicators, and optics—have to work together. It’s a very impressive synergy, but still very young when you think about it.

Nozomi Nishimura: Well, that’s exciting. That’s good to hear. That’s fantastic.

Any last advice? Anything you want to let the neurophotonics community know?

David Kleinfeld: Stay friendly and be critical. That sounds glib, but I think it’s very important. First of all, you may work very hard at something, you might not want to share something until you get it out, but I honestly have found—I think you and Chris work this way, too—that you tell people things, you help them, and people help you. Right? I mean, you don’t want to get stuck and find out that you could’ve talked to somebody and not spent three months solving a problem, because somebody else knows how to do it. So, I think it’s very important to share, in a critical way.

I also think it’s very important to be critical in your evaluation of data. Physicists are fundamentally critical. I went to one APS meeting in my life, and I was cracking up—I went to an EXAFS section, on x-ray structure—and somebody puts up their hand and they go “Regarding that alleged data that you just showed”—and I thought, this is hilarious! Maybe that was a little bit the Bell Laboratories way, but biologists are much too friendly—you know, “Congratulations on your lovely data!”—I mean, you have to stay friends with people, but you have to be extremely critical. If something doesn’t seem right, you can’t let it pass, because if things get into the literature that aren’t done well, that are somewhat wrong, then basically the field will grind to a halt because it will pick up booboos.

I think you could see this in the historical record. In the 1980s, and in the 70s, there was very impressive work on circuitry in small systems, and there were some apparently very smart people doing this. And it kind of ground to a halt. When you look back at this—and I’ve done this a little bit, because these are examples you want to use in your classes—it seems that people were working on different animals to avoid stepping on toes. And they were afraid to criticize each other. Booboos got into the literature, and the field of “simple nervous systems” mostly has halted. And then there just wasn’t a lot of circuitry work when you get down to it. It’s only now, after all this fancy technology. Right? There was this dead time of like 20 years, 30 years. And all of a sudden, maybe mostly through zebrafish, where you can image through the whole fish, people are beginning to form completed circuits again. It’s a little more of a competitive world, so I’m not sure if booboos are going to build up again without being challenged, but I think that’s something we just have to be on our guard against. Right?

You can still be friends with people, and you can still point out things that don’t make sense. And then the field as a whole will thrive. Words of wisdom or words of age, or something like that. Again, if it were sports, you wouldn’t think twice about it. If you’re on a swim team and your colleague has something wrong about their stroke, you would just be all over them and point it out. Because, you know, you want to win the relay. Right?

Nozomi Nishimura: That’s a good analogy.

David Kleinfeld: So, I think we have to do the same in science.

Nozomi Nishimura: Sounds good! Well, thanks very much. That was a lot of fun and we’ll hope to maybe see each other in person at a conference or something soon.

